# Comparison of anthropometric data quality in children aged 6-23 and 24-59 months: lessons from population-representative surveys from humanitarian settings

**DOI:** 10.1186/s40795-020-00385-0

**Published:** 2020-11-13

**Authors:** Oleg Bilukha, Alexia Couture, Kelly McCain, Eva Leidman

**Affiliations:** 1 Emergency Response and Recovery Branch, Division of Global Health Protection, Center for Global Health, Centers for Disease Control, 1600 Clifton Road, Atlanta, GA 30329 USA; 2 Rollins School of Public Health, Emory University, Atlanta, USA

**Keywords:** Anthropometry, Age group, Data quality, Survey, Humanitarian

## Abstract

**Background:**

Ensuring the quality of anthropometry data is paramount for getting accurate estimates of malnutrition prevalence among children aged 6–59 months in humanitarian and refugee settings. Previous reports based on data from Demographic and Health Surveys suggested systematic differences in anthropometric data quality between the younger and older groups of preschool children.

**Methods:**

We analyzed 712 anthropometric population-representative field surveys from humanitarian and refugee settings conducted during 2011–2018. We examined and compared the quality of five anthropometric indicators in children aged 6–23 months and children aged 24–59 months: weight for height, weight for age, height for age, body mass index for age and mid-upper arm circumference (MUAC) for age. Using the z-score distribution of each indicator, we calculated the following parameters: standard deviation (SD), percentage of outliers, and measures of distribution normality. We also examined and compared the quality of height, weight, MUAC and age measurements using missing data and rounding criteria.

**Results:**

Both SD and percentage of flags were significantly smaller on average in older than in younger age group for all five anthropometric indicators. Differences in SD between age groups did not change meaningfully depending on overall survey quality or on the quality of age ascertainment. Over 50% of surveys overall did not deviate significantly from normality. The percentage of non-normal surveys was higher in older than in the younger age groups. Digit preference score for weight, height and MUAC was slightly higher in younger age group, and for age slightly higher in the older age group. Children with reported exact date of birth (DOB) had much lower digit preference for age than those without exact DOB. SD, percentage flags and digit preference scores were positively correlated between the two age groups at the survey level, such as those surveys showing higher anthropometry data quality in younger age group also tended to show higher quality in older age group.

**Conclusions:**

There should be an emphasis on increased rigor of training survey measurers in taking anthropometric measurements in the youngest children. Standardization test, a mandatory component of the pre-survey measurer training and evaluation, of 10 children should include at least 4–5 children below 2 years of age.

## Background

Parameters for assessing anthropometric data quality, put forth by a World Health Organization (WHO) Expert Committee in 1995 and expanded on in a 2019 report by a WHO-UNICEF (United Nations Children’s Fund) Technical Expert Advisory Group on Nutrition Monitoring (TEAM), are well established [1, 2]. Six key parameters are recommended: standard deviation of z-score distribution, evaluation of missing data, sex ratios, digit preference, outlier values, and measures of the shape of z-score distribution. Standard deviation (SD) of z-score distribution is a key metric of measurement error because the introduction of random error results in a widening of the observed distribution. Absent measurement error, distributions are expected to be approximately normal with a SD close to 1 [1, 2]. Wider SDs result in an artificial inflation in prevalence of nutritional indicators derived from converting a continuous variable [such as z-score or body mass index (BMI) value] into categorical using defined cut-off values [3, 4]. Prior research has demonstrated independence between the nutritional status of a population, as measured by the mean z-score value, and standard deviation [5, 6].

Assessing completeness of the weight, height, age and sex variables is recommended since missing data may bias nutritional indicators if data are not missing at random. Similarly, current guidelines recommend assessing sex ratios and age distributions as an imbalance in the sample that does not represent the underlying population in terms of these basic demographic parameters could also indicate selection bias. Measures of digit preference for age, height, weight and MUAC (if collected) are recommended to evaluate whether there are indications of possible rounding [2]. The proportion of biologically or statistically implausible z score values is routinely reported [1]. These outlier values, commonly referred to as ‘flags’, are reviewed to identify measurements that are likely to be errors. Often these extreme values are the result of data entry mistakes, such as those occurring when a date of birth is recorded as March 8 rather than August 3. Finally, evidence of non-normal distributions (as indicated by significant skewness and kurtosis) may indicate poorer age ascertainment or anthropometric data quality.

In anthropometric field surveys the above parameters are calculated for the entire sample of children 6–59 months of age for whom anthropometric measurements were collected [7]. Assessment of anthropometric data quality by age group is not a routine practice. However, age-related differences in difficulty of measurement are well recognized, and noted in training manuals and manuscripts [5, 7–9]. For example, measurement of standing height in children aged above 2 years is commonly thought to be easier than measurement of recumbent length in younger children. Children often resist being positioned in the supine position; resistance makes obtaining an accurate measurement a challenge. The reverse is observed for ascertainment of age. In settings where vital registration is poor and celebrating birthdays is not part of the culture, prompting caregivers to recall child’s age using a local events calendar may be simpler for younger children with more recent dates of birth.

In a technical report evaluating anthropometric data quality, the Demographic and Health Survey (DHS) program assessed differences in standard deviation of z-scores by age (under 2 years vs. those aged 2–4 years) for measures of weight for height (WHZ) and height for age (HAZ). Of 52 country surveys included in their analysis, the SDs of both HAZ and WHZ measurements were higher for children under 2 years old than for children 2 to 4 years old for all countries except Armenia [9]. The impact of these field challenges on the quality of other derived indicators – weight-for-age (WAZ), mid-upper arm circumference for age (MUACZ), and body mass index for age (BMIZ) – have not been characterized. Similarly, the DHS analysis evaluated only SD; age differences in quality of other indicators have not been formally explored.

For this study we characterized differences in age ascertainment and anthropometric quality for younger children 6–23 months compared to older children 24–59 months, using standard criteria based on the z-score distribution of each indicator (SD, percentage of outliers, and measures of distribution normality) as well as the absolute values of height, weight, MUAC and age measurements (missing data and rounding). The work takes a broad focus, evaluating five anthropometric indicators (weight for height, height for age, weight for age, MUAC for age, and BMI for age) as well as all recommended parameters of data quality. A secondary aim was to explore whether the difference in age ascertainment and anthropometric quality comparing older and younger children depend on the anthropometric status of the population or overall quality of the survey.

## Methods

Data for these analyses were single stratum, cross-sectional, population representative anthropometric surveys generally conducted at the district, sub-district or refugee camp level (referred to as “small-scale surveys”) provided by the United Nations High Commissioner for Refugees (UNHCR) and by Action Contre la Faim (ACF). Surveys were conducted between 2011 and 2018 and measured age, sex, weight, height and MUAC in children aged 6–59 months following standard procedures and using questionnaires developed from a standard template [7, 10]. Surveys sampling designs were either two-stage cluster, exhaustive, or simple random. Weight-for-height (WHZ), Weight-for-age (WAZ), Height-for-age (HAZ), MUAC-for-age (MUACZ), and BMI-for-age (BMIZ) z-scores were calculated for all children using the WHO SAS macro, which applies the WHO 2006 growth standards [11].

Surveys were included if they measured a minimum set of anthropometric indicators: sex, age, height and weight. MUAC measurement was not required for inclusion. Surveys with sample size smaller than 200 persons were excluded given concerns about adequate precision; surveys with fewer than 200 persons would have precision greater than 30% of the estimated prevalence of global acute malnutrition, as proposed by Prudhon et al., for surveys with prevalence ranges observed in our dataset [12]. Surveys with greater than 1200 persons were excluded given an interest in small-scale emergency surveys. Cluster surveys with fewer than 25 clusters or those missing cluster identification variable for more than 20% of sampled children were also excluded from analysis. Additionally, a survey with an implausible ratio of males to females (> 125:1) was excluded. To exclude surveys with exceptionally poor anthropometry data quality or where data manipulation might be suspected, we excluded from analysis surveys where the SD for WHZ, WAZ, HAZ, or BMIZ was outside of the following empirically defined cutoffs: greater than 1.8 or lower than 0.8; or the SD for MUACZ greater than 1.8 and less than 0.7. These cutoffs correspond approximately to the 1st and 99th percentiles of distributions in our dataset. Finally, surveys for which more than 20% of children were missing MUAC measurements were excluded from analysis of MUACZ but kept for analyses of other indicators.

Within surveys retained for analysis, children were excluded if the child record was an exact duplicate of another for all variables including identifier variables. Children with missing data for sex, weight, height or MUAC were excluded by indicator, such that a child with missing weight would be excluded from WHZ but not MUACZ calculations. Outlier observations (“flagged values”) were excluded from a survey if z-score of a child fell outside the flexible exclusion range of +/− 4 z-scores from the observed survey sample mean, as described by WHO [1]. Those outliers were also excluded by indicator.

Standard, survey level measures of data quality were computed: standard deviation, percent of children flagged, skewness, excess kurtosis, and normality of the distribution (using Shapiro-Wilk test) for all five anthropometric indicators (WHZ, WAZ, HAZ, MUACZ, and BMIZ). Digit preference score was calculated for weight, height, MUAC, and age category applying the MONICA procedure such that a value of zero indicates a uniform distribution and values increase with greater imbalance [13]. Digit preference for age was calculated on binned data such that age in months was binned into six-month groups given expected pattern of heaping in which age is rounded to the nearest year and half-year. All of these computed survey level variables were produced for the sample overall (e.g., all children 6–59 months), as well as separately for two age categories of children, 6–23 months and 24–59 months.

First we evaluated quality of age ascertainment and the measured anthropometric variables (weight, height, and MUAC). For each variable, we calculated the medians of survey level digit preference (DP) scores for the sample overall, and separately for younger and older children. We tested the median difference between younger and older age groups with non-parametric tests. Spearman correlations were calculated to examine the correlation between DP of younger and older age groups. Percentages of surveys where younger children had better (lower) DP scores were calculated. A final analysis was performed to explore how ascertainment of age impacted quality. For this analysis, the pooled data (ignoring survey level) was evaluated. Children were categorized as having a date of birth (DOB) or estimated age in months and DP scores for age category were calculated for children with DOB recorded versus children without birthday recorded. We also report the mean percent of missing data for weight, height, MUAC, and exact birthday, overall and by age group.

To examine whether data quality differed meaningfully for younger and older children, we present the mean of survey level estimates of all continuous measures of data quality mentioned above for the sample overall, as well as for children 6–23 months, and for children 24–59 months. For the continuous measures of data quality, we calculated the mean of the means differences between the younger children and older children within each survey and tested if these differences were significant using a paired t-test. Pearson correlations were calculated to describe correlations between the paired values from two age groups. We calculated the percent of surveys where younger children had better quality (i.e. lower SD, lower percent of flagged children, absolute value of skewness and excess kurtosis closer to zero). These analyses were done for each of the five anthropometric indicators. Additionally, we defined cutoffs for skewness as +/− 0.5 and excess kurtosis as +/− 1 [2]. For both skewness and excess kurtosis, we calculated the percent of surveys outside of those cutoffs for all children, as well as younger and older age groups, indicating the percent of those that were negative and those that were positive. Finally, we calculated the percent of surveys that were non-normal as defined by a *p*-value < 0.05 of the Shapiro-Wilk test for all children, younger and older, and the difference of this proportion between younger and older groups.

To evaluate whether the difference in standard deviation comparing younger and older children depended on the quality of age ascertainment, we categorized the surveys based on percent of children in a given survey with exact DOB recorded. For this analysis, we categorized the surveys into those with greater than 90% of children with DOB recorded, between 10 and 90% of children with DOB recorded, and less than 10% of children with DOB recorded. We repeated the SD analysis as before for these three categories.

This study was determined not to be human subjects research by the institutional review board of the Centers for Disease Control and Prevention as it entailed secondary analysis of routinely collected programmatic data. No individual identifiers were included in the dataset used for analysis. Data were aggregated, analyzed and visualized using SAS Version 9.4 and RStudio [14, 15].

## Results

**Fig. 1:**
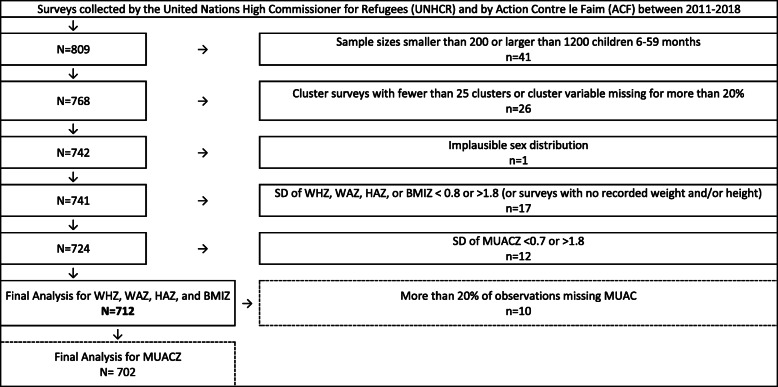
Included surveys among all surveys collected by the United Nations High Commissioner for Refugees (UNHCR) and by Action Contre la Faim (ACF) between 2011 and 2018

Data from 809 anthropometric surveys conducted by ACF or UNHCR between 2011 and 2018, that measured sex, age, weight, and height in children aged 6–59 months, were considered for analysis. Of them, 97 met exclusion criteria (Fig. 1). The remaining 712 surveys were retained for further analysis. Of the 712, 702 also measured MUAC. The surveys were conducted in 41 countries, regional origin of retained surveys is shown in Supplementary File. A total of 383,589 children aged 6–59 months were measured in these 712 surveys, of which 35.9% were children aged 6–23 months (Supplementary File). Data on weight, height, and sex were missing for 0.63, 0.47, and < 0.01% of children, respectively. Among children included in the 702 surveys that measured MUAC (*n* = 379,169), MUAC was missing for 0.52%.

**Fig. 2:**
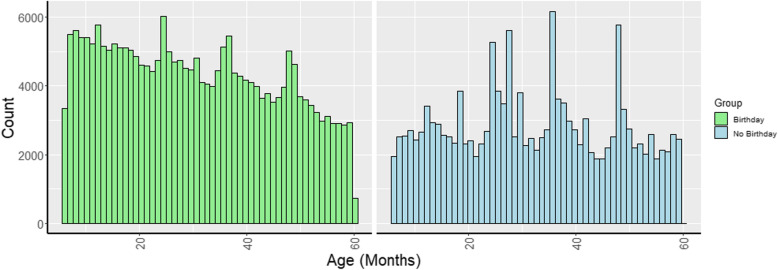
Age distribution by month among children aged 6–59 months who do and do not have exact date of birth in combined sample from 712 field surveys. Categories represented by colors are as follows: Children with exact date of birth recorded (green), children with only year and month of birth recorded (blue)

Quality of height, weight, MUAC and age measurements was compared for the two age groups based on the proportion of children with missing values and on digit preference score (Table 1). The proportion of children missing weight, height, and MUAC were all less than 1%, such that differences by age group were not meaningful. Overall, percentage of children per survey missing exact date of birth was 37.3%. The proportion of children missing date of birth was higher among older children (39.9% vs 32.6%, *p* < 0.001) (Table 1, Fig. 2). Mean digit preference for weight (8 vs 6), height (10 vs 8) and MUAC (9 vs 7) were all significantly higher among younger children (*p* < 0.001 for all measurements); the opposite was true for age (9 vs 8). Difference in digit preference between children with and without DOB (not stratified by age group), was pronounced. Children with complete date of birth were much less likely to have age rounded to the nearest 6-month intervals than children for which age was estimated in months (Fig. 2), indicated by much lower digit preference scores (7 v. 16) (Table 1). Digit preference scores for all anthropometric measurements were significantly correlated such that surveys in which more rounding was observed among younger children also had higher digit preference scores among older children (correlation range: 0.26–0.60).

**Table 1:** Evaluation of quality of height, weight, mid-upper arm circumference and age using missing values and digit preference, scores overall and by age group

Indicator		Monica DP (median)	% Missing (survey level mean)	% Missing (overall dataset)
Weight	Children 6–59 m	5	0.6%	0.6%
	Children 6–23 m	8	0.6%	0.6%
	Children 24–59 m	6	0.6%	0.6%
	Difference^a^	2***	–	–
	Correlation^b^	0.4***	–	–
	% younger^c^ better	13.6%	–	–
	% older^c^ better	71.5%	–	–
Height	Children 6–59 m	8	0.5%	0.5%
	Children 6–23 m	10	0.5%	0.5%
	Children 24–59 m	8	0.5%	0.5%
	Difference^a^	2***	–	–
	Correlation^b^	0.6***	–	–
	% younger^c^ better	19.1%	–	–
	% older^c^ better	69.2%	–	–
MUAC	Children 6–59 m	6	0.5%	0.5%
	Children 6–23 m	9	0.6%	0.7%
	Children 24–59 m	7	0.5%	0.4%
	Difference^a^	2***	–	–
	Correlation^b^	0.6***	–	–
	% younger^c^ better	16.4%	–	–
	% older^c^ better	70.8%	–	–
Age Category	Children 6–59 m	7	37.3%	39.2%
	Children 6–23 m	8	32.6%	34.1%
	Children 24–59 m	9	39.9%	42.1%
	Difference^a^	−1***	–	–
	Correlation^b^	0.3***	–	–
	% younger^c^ better	51.0%	–	–
	% older^c^ better	40.7%	–	–
	With birthdays	7	–	–
	Without birthdays	16	–	–
	Difference^d^	−10***	–	–

DP Digit preference; MUAC Mid-upper arm circumference; SD Standard deviation

% missing MUAC excluded surveys excluded from MUAC analysis

% missing for age category is % missing birthday/exact DOB

*P*-values were calculated using non-parametric tests

*p < 0.05; **p < 0.01; ***p < 0.001

^a^ Difference between 6–23 and 24–59 months age groups

^b^Correlation between 6–23 and 24–59 age groups

^c^ ‘Younger better’ refers to the percentage of surveys where absolute values for age group 6–23 months were lower that absolute values for age group 24–59 months. ‘Older better’ refers to the percentage of surveys where absolute values for age group 6–23 months were greater that absolute values for age group 24–59 months. Surveys in which the values in the two age groups were equivalent not included in either figure

^d^Difference between sub-set of children with exact date of birth and children for whom age was estimated to the nearest month

**Fig. 3:**
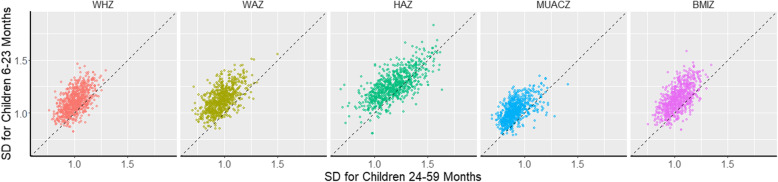
Scatterplots of standard deviations of anthropometric indicator among sub-samples of younger (6–23 months) and older (24–59 months) children in surveys of children 6–59 months, by indicator. SD: Standard deviation; WHZ: Weight-for-height z-score; WAZ: Weight-for-age z-score; HAZ: Height-for-age z-score; MUACZ: Mid-upper arm circumference-for-age z-score; BMIZ: Body mass index-for-age z-score. Dotted lines satisfy x = y as a reference

Means of the survey level z-score standard deviations by anthropometric indicator and by age group are presented in Table 2. For all five indicators, the mean standard deviation by survey among younger children was greater than that of older children: WHZ (1.11 v. 1.07), WAZ (1.13 v. 0.98), HAZ (1.26 v. 1.18), MUACZ (1.02 v. 0.91), BMIZ (1.14 v. 1.04). The difference in z-score standard deviation among children 6–23 months compared to children 24–59 months from the same survey was significant (*p* < 0.001) for all indicators. The greatest mean difference was observed for WAZ (0.15) and the smallest for HAZ (0.08). Percentage of surveys where younger children had smaller SD than older children ranged from 5.5% (for WAZ) to 25.6% (for HAZ). A similar pattern was observed for the proportion of children flagged as outliers, where the mean proportion of younger children flagged was significantly higher than that of older children (*p* < 0.001) for all indicators. For both SD and percentage of flags, observed values among younger children and older children were significantly positively correlated for all five anthropometric indicators (Table 2, Fig. 3). Correlations for standard deviation ranged from 0.56 (WHZ) to 0.69 (HAZ). Correlations for percent flags ranged from 0.19 (MUACZ) to 0.82 (BMIZ).

**Table 2:** Evaluation of quality parameters (standard deviation, percentage of flags, skewness, excess kurtosis, normality) for anthropometric indices among children 6–59 months, overall and by age group

Indicator		SD (mean)	% Flag (mean)	Skewness (mean)	% Skewed (+/− 0.5 cutoffs)	% Skewed negative	% Skewed positive	Kurtosis (mean)	% Kurtosis (+/− 1 cutoffs)	% excess kurtosis negative	% excess kurtosis positive	% Non-Normal (Shapiro)
WHZ	Children 6-59 m	1.05	0.36	− 0.09	1.9%	1.4%	0.5%	0.30	3.0%	0.0%	3.0%	45.7%
	Children 6–23 m	1.11	0.49	−0.05	3.1%	1.8%	1.3%	0.17	4.1%	0.0%	4.1%	16.0%
	Children 24–59 m	1.07	0.28	−0.07	3.4%	2.3%	1.1%	0.36	9.6%	0.0%	9.6%	38.3%
	Difference^a^	0.10***	0.21***	0.01	−0.3%	–	–	− 0.18***	−5.5%***	–	–	−22.3%***
	Correlation^b^	0.56***	0.40***	0.30***	–	–	–	0.18***	–	–	–	–
	% younger^c^ better	14.3%	21.9	51.0%	–	–	–	54.8%	–	–	–	–
WAZ	Children 6–59 m	1.04	0.19	−0.07	1.4%	1.3%	0.1%	0.27	3.7%	0.0%	3.7%	42.4%
	Children 6–23 m	1.13	0.33	−0.03	3.8%	2.0%	1.8%	0.13	3.9%	0.0%	3.9%	13.6%
	Children 24–59 m	0.98	0.09	−0.12	4.4%	3.8%	0.6%	0.28	9.0%	0.0%	9.0%	35.8%
	Difference^a^	0.15***	0.24***	0.09***	−0.6%	–	–	− 0.15***	−5.0%***	–	–	−22.2%***
	Correlation^b^	0.59***	0.23***	0.15***	–	–	–	0.17***	–	–	–	–
	% younger^c^ better	5.5%	10.4%	52.8%	–	–	–	50.6%	–	–	–	–
HAZ	Children 6–59 m	1.22	1.00	0.03	1.1%	0.4%	0.7%	0.11	2.5%	0.0%	2.5%	38.9%
	Children 6–23 m	1.26	1.52	0.03	3.7%	1.1%	2.6%	0.05	1.4%	0.0%	1.4%	17.3%
	Children 24–59 m	1.18	0.67	0.01	3.7%	2.0%	1.7%	0.14	3.9%	0.0%	3.9%	33.9%
	Difference^a^	0.08***	0.86***	0.02*	0.0%	–	–	−0.10***	−2.5%***	–	–	−16.6%***
	Correlation^b^	0.69***	0.75***	0.30***	–	–	–	0.31***	–	–	–	–
	% younger^c^ better	25.6%	14.5%	47.9%	–	–	–	51.8%	–	–	–	–
MUACZ	Children 6–59 m	0.96	0.13	−0.11	3.6%	3.3%	0.3%	0.32	28.2%	0.3%	27.9%	41.0%
	Children 6–23 m	1.02	0.17	−0.13	9.0%	8.6%	0.3%	0.31	32.3%	3.4%	28.9%	26.5%
	Children 24–59 m	0.91	0.10	−0.11	5.8%	5.4%	0.4%	0.26	26.1%	1.6%	24.5%	32.2%
	Difference^a^	0.10***	0.08***	−0.03*	3.1%*	–	–	0.05*	6.3%*	–	–	−5.7%*
	Correlation^b^	0.58***	0.19***	0.25***	–	–	–	0.06	–	–	–	–
	% younger^c^ better	10.4%	15.5%	42.0%	–	–	–	43.0%	–	–	–	–
BMIZ	Children 6–59 m	1.08	1.56	−0.09	1.3%	0.8%	0.5%	0.29	26.4%	0.4%	26.0%	43.3%
	Children 6–23 m	1.14	2.14	−0.08	3.8%	3.0%	0.8%	0.16	23.7%	4.6%	19.1%	15.7%
	Children 24–59 m	1.04	1.24	−0.05	3.0%	1.5%	1.5%	0.35	35.1%	0.8%	34.3%	34.3%
	Difference^a^	0.10***	0.89***	−0.03**	0.8%	–	–	−0.19***	−11.4%***	–	–	−18.5%***
	Correlation^b^	0.63***	0.82***	0.30***	–	–	–	0.18***	–	–	–	–
	% younger^c^ better	16.4%	25.8%	51.8%	–	–	–	56.2%	–	–	–	–

WHZ Weight-for-height z-zcore, WAZ Weight-for-age z-score, HAZ Height-for-age z-score, MUACZ Mid-upper arm circumference-for-age z-score, BMIZ BMI-for-age z-score, SD Standard deviation

*P*-values were calculated using parametric tests

*p < 0.05; **p < 0.01; ***p < 0.001

^a^ Mean difference between 6–23 and 24–59 months age groups

^b^ Correlation between 6–23 and 24–59 age groups

^c^Percentage of surveys where absolute values for age group 6–23 months were lower than absolute values for age group 24–59 months

Differences in SD stratified by quality of age ascertainment (as evaluated by the proportion of children with exact DOB) are presented in Table 3. For WHZ and BMIZ, SD differences between younger and older children were greater for surveys with better age ascertainment, however the opposite was true for HAZ, and no clear trend was observed for WAZ and MUACZ.

**Table 3:** Evaluation of standard deviation of anthropometric indices overall and by age group, by the quality of age ascertainment (based on percentage of children with exact date of birth)

Indicator		Birthday > 90% (mean)	Birthday 10–90% (mean)	Birthday < 10% (mean)
WHZ	N	272	309	131
	Children 6–59 m	1.03	1.06	1.08
	Children 6–23 m	1.08	1.11	1.15
	Children 24–59 m	0.99	1.02	1.03
	Difference^a^	0.09***	0.10***	0.12***
	Correlation^b^	0.62***	0.51***	0.53***
	% younger^c^ better	5.2%	6.9%	2.3%
WAZ	N	272	309	131
	Children 6–59 m	1.02	1.05	1.05
	Children 6–23 m	1.10	1.14	1.14
	Children 24–59 m	0.96	0.99	0.99
	Difference^a^	0.15***	0.15***	0.15***
	Correlation^b^	0.64***	0.55***	0.53***
	% younger^c^ better	2.0%	3.0%	0.6%
HAZ	N	272	309	131
	Children 6–59 m	1.17	1.24	1.27
	Children 6–23 m	1.21	1.28	1.29
	Children 24–59 m	1.13	1.20	1.24
	Difference^a^	0.09***	0.08***	0.06***
	Correlation^b^	0.68***	0.69***	0.64***
	% younger^c^ better	8.3%	11.5%	5.8%
MUACZ	N	264	307	131
	Children 6–59 m	0.95	0.97	0.97
	Children 6–23 m	1.00	1.02	1.04
	Children 24–59 m	0.90	0.92	0.92
	Difference^a^	0.10***	0.10***	0.12***
	Correlation^b^	0.62***	0.55***	0.57***
	% younger^c^ better	4.4%	4.8%	1.1%
BMIZ	N	272	309	131
	Children 6–59 m	1.05	1.09	1.12
	Children 6–23 m	1.10	1.14	1.18
	Children 24–59 m	1.01	1.05	1.06
	Difference^a^	0.09***	0.10***	0.12***
	Correlation^b^	0.68***	0.54***	0.61***
	% younger^c^ better	5.9%	8.0%	2.5%

WHZ Weight-for-height z-score, WAZ Weight-for-age z-score, HAZ Height-for-age z-score, MUACZ Mid-upper arm circumference-for-age z-score, BMIZ BMI-for-age z-score, SD Standard deviation, m months

*P*-values were calculated using parametric tests

*p < 0.05; **p < 0.01; ***p < 0.001

^a^Difference between 6–23 and 24–59 months age groups

^b^ Correlation between 6–23 and 24–59 age groups

^c^ Percentage of surveys where absolute values for age group 6–23 months were lower that absolute values for age group 24–59 months

**Fig. 4:**
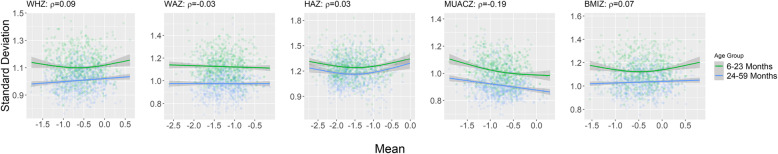
Correlation of standard deviations and mean of anthropometric indicator in surveys of children 6–59 months, by indicator and age group. SD: Standard deviation; WHZ: Weight-for-height z-score; WAZ: Weight-for-age z-score; HAZ: Height-for-age z-score; MUACZ: Mid-upper arm circumference-for-age z-score; BMIZ: Body mass index-for-age z-score. Spearman correlations for 6–59 months age group represented by ρ

To explore whether the z-score SD overall and by age group were independent of survey’s z-score mean, we plotted the SD against the mean (Fig. 4) for all five anthropometric indicators. For WHZ, HAZ, WAZ, and BMIZ for the sample overall, as well as for younger and older children, correlations were negligible (all below 0.1). Correlation coefficients ranged from − 0.03 to 0.09 for the sample overall, from − 0.03 to 0.03 for younger children, and from 0 to 0.10 for older children. For MUACZ, a larger negative correlation was observed for both the sample overall (*ρ* = − 0.19) and for younger (*ρ* = − 0.20,) and older children (*ρ* = − 0.17). For all parameters, the difference in standard deviations comparing younger and older children are independent of the anthropometric status of the population as indicated by the mean z-score (Fig. 4).

The majority of surveys had distributions that were normal, as evaluated by Shapiro-Wilk test, for all five anthropometric indicators (Table 2). The proportion of surveys with z-scores that were normally distributed was lowest for WHZ (54.4%) and highest for HAZ (61.1%). Skewness was out of the +/− 0.5 range for less than 4% of surveys for all indicators whereas excess kurtosis was out of the +/− 1.0 range for less than 4% surveys for WHZ, WAZ and HAZ but the proportion of kurtotic surveys was notably higher for MUACZ (28.2%) and BMIZ (26.4%). Surveys with excess kurtosis out of range were nearly all positively kurtotic. The proportion of surveys with skewness out of range was not significantly different for younger and older children for any indicator. The proportion of surveys with excess kurtosis out of range was significantly higher for older children for WHZ (4.1% v. 9.6%), WAZ (3.9% v. 9.0%), HAZ (1.4% v. 3.9%) and BMIZ (23.7% v. 35.1%) but lower for older children for MUACZ (32.3% v. 26.1%). Differences in mean excess kurtosis values as well as the proportion of surveys out of range for excess kurtosis comparing younger and older children were significant for all indicators (*p* < 0.05 for MUACZ, and *p* < 0.001 for WHZ, WAZ, HAZ, BMIZ).

## Discussion

The primary finding of our analysis is that the quality of all five anthropometric indicators investigated was consistently and significantly higher in older (24–29 months) than younger (6–23 months) age group, based on two key quality parameters, SD of z-scores and percentage of flags. This is fully in line with previous reports. Analysis of 51 DHS surveys published in 2007 found that the quality of measurements as assessed by SD was higher in age group 36–59 months compared to age group 0–35 months for WHZ, HAZ, WAZ and BMIZ [5]. More recent in-depth quality assessment of 52 DHS surveys conducted between 2005 and 2014 reported that SD of WHZ and HAZ were narrower in age group 24–59 months compared to age group 0–23 months in all 52 analyzed surveys for HAZ and in all but one survey for WHZ [9]. Those analyses did not report comparisons using percentage of flags and did not investigate comparisons based on MUACZ since MUAC measurements are not included in DHS surveys. An important nuance however is that our analysis found that for a sizeable proportion of surveys this difference does not hold – for example, whereas the DHS report found that HAZ SD for all of 52 surveys analyzed was narrower in the older age group, our analysis demonstrated that the opposite was true (younger group had a narrower SD) in about a quarter of the 712 small-scale surveys [9]. Further, the differences in SD between age groups observed in our analysis for WHZ and HAZ (0.10 and 0.08, respectively) were much smaller than those reported for WHZ and HAZ (0.24 and 0.29, respectively) in the 2015 DHS data quality report.

The magnitude of differences in SD between age groups was the highest for WAZ and the lowest for HAZ. These differences in SD did not change meaningfully or show a consistent trend when surveys were classified based on the quality of age ascertainment (based on percentage of children with available DOB). Further, the anthropometric quality overall and the differences in quality between older and younger age groups did not depend on the nutrition status of the survey population (as described by mean z-score). The only indicator for which we observed a consistent, but relatively small, improvement in quality (narrowing of SD) with improving anthropometric status (increasing mean z-score) was MUACZ.

Notably, overall quality, as assessed by SD, was highest for MUACZ (0.96) and lowest for HAZ (1.22). The average SD for the remaining indicators (WHZ, HAZ and BMIZ) were in the 1.0–1.1 range. The lowest quality of HAZ could be ascribed to difficulties with age ascertainment and height measurement. However, interestingly, the same can be argued as true for MUACZ – age is equally hard to ascertain, and there are challenges with measuring MUAC, and yet MUACZ is showing the highest quality of measurements among the five indicators. The reasons for this are not well understood and merit further inquiry. As expected, for all indicators the overall quality, as ascertained by SD, was higher in surveys where > 90% of children had an exact DOB compared to the surveys where less than 10% had exact DOB, but perhaps this difference in quality was not as pronounced as expected. The overall difference in SD between surveys with very high and very low DOB ascertainment was the highest for HAZ (0.10), with the other four indicators showing much smaller differences (0.03–0.05).

The second important finding was that the two key quality parameters (SD and percentage of flags) were significantly positively correlated at the survey level between young and old age groups, such that surveys having higher quality of measurements in younger age group also tended to have higher quality in older age group. This is intuitively expected, as the surveys where measurers are thoroughly trained, motivated and supervised would tend to produce higher quality of data irrespective of child age [8].

The second large battery of analyses we performed investigated normality of z-score distributions, overall and by age group, using the cutoffs for skewness (+/− 0.5) and excess kurtosis (+/− 1.0) proposed in the recent WHO surveys guidelines [2]. We discovered that overall percentage of skewed survey distributions was very small – 3.6% for MUACZ and less than 2% for the remaining four indicators, and that the differences in proportions of skewed distributions between age groups were small and not meaningful for practical purposes. Overall proportion of kurtotic surveys was low (< 4%) for three of the five indicators (WHZ, HAZ and WAZ). However, for MUACZ and BMIZ the proportion of kurtotic surveys exceeded 25%. The reasons for this are not immediately clear and require further investigation. Moreover, we noted that almost all kurtotic distributions we identified had positive rather than negative excess kurtosis, in lay terms meaning that the relative balance of the size of body vs. tails of the distribution was shifted towards the larger (than expected for normal distribution) tails and smaller body. It has to be noted that excess kurtosis of the distribution is highly dependent on the range of flags exclusion, such as the narrower ranges of exclusion (for example +/− 3 z-scores from survey mean) used in SMART survey quality checks would exclude more outliers from the tails and thus result in relatively smaller tails and more negative excess kurtosis statistic than wider exclusion ranges (+/− 5 or +/− 6 z-scores from the reference mean, depending on indicator) used in DHS and MICS surveys. As noted in the recent WHO survey guidelines [2], defining the optimal range for flags exclusion requires further investigation, and analysis of excess kurtosis produced by different flag exclusion ranges may be instrumental in this pending work. The majority of surveys (54–61%, depending on indicator) overall had no significant deviation from normality of their z-score distribution as assessed by Shapiro-Wilk test. This proportion was consistently higher for the younger than older age group, however this should be interpreted with caution. As noted by the authors of the recent study investigating normality of MUAC distributions, significance of Shapiro-Wilk test is dependent on the sample size, such that smaller sample sizes are less likely to produce significant Shapiro-Wilk test results [16]. Since the sample sizes were approximately two times smaller for younger compared with older age group given the narrower age range, the paucity of significant test results in younger group may be to some extent driven by smaller sample sizes.

Finally, we investigated the quality of individual anthropometric measurements (weight, length/height, MUAC and age) using missing value and rounding criteria. Proportions of missing values for height, weight, and MUAC were trivial (around 0.5%) with no significant differences between the age groups. Approximately two out of five children overall had no exact DOB. In line with the assumption that vital registration is improving over time, the proportion of younger children with no exact DOB was slightly lower as compared with older age group (32.6% vs. 39.9%). As expected, given more challenges in taking anthropometric measurements in youngest children, we found small but significant preponderance to rounding of weight, height, and MUAC in younger as compared to older age group (for all measures the difference of 2 units of digit preference score). Age rounding was slightly less problematic in younger children, in line with observed higher proportion of children with exact DOB in this age group. As expected, age rounding was much higher in the pooled group of children with no exact DOB compared with the children with exact DOB (digit preference scores of 16 and 7, respectively); the magnitude of the effect of estimating age where DOB is unknown has not been well characterized previously. Similar to what was noted for SD and percentage flags, digit preference scores were also significantly positively correlated for all measures (weight, height, MUAC and age) between young and old age groups, such that surveys showing less rounding in the younger age group were also showing less rounding in older age group.

A major strength of this study is the high number and ascertained quality of the cross-sectional surveys it builds upon as well as the fact that all these surveys were conducted in the last 10 years and therefore best reflect current field practices. The 712 surveys contributing to this analysis were conducted in 41 countries around the world. During these surveys, planning, data collection, and analysis followed standardized methods embedding rigorous quality controls and were supervised and validated a posteriori by highly qualified and trained staff [7, 10]. This study however has several notable limitations. First, only surveys from humanitarian or refugee settings are included. Further, the surveys were small scale, generally with the objective of providing anthropometric estimates at the district or refugee settlement level. Thus, the results we obtained may not be representative of the overall countries or regions. Second, we did not have detailed documentation of the anthropometric equipment used in these surveys. While Shorr boards for length/height and tapes for MUAC used in these surveys are very similar or identical across settings, the difference in weight measurement devices used (electronic floor scales versus spring hanging “pants” scales) may have been important and may have affected the quality outcomes for weight-dependent indicators (WHZ and WAZ). Within a survey, young and older children are measured using the same equipment. Lastly, this analysis did not include children aged 0–5 months, since this age group is not routinely included in small scale anthropometric surveys in humanitarian settings. The difference in quality observed between age groups in this analysis therefore should be extrapolated with caution to this youngest age group, although the DHS analyses described above did include this age group and demonstrated results fully in line with ours.

## Conclusion

This study expands on limited available evidence to include additional indicators (notably MUACZ) and multiple additional quality parameters beyond SD used in previous reports. It shows not only consistently higher quality of anthropometric data among children aged 24–59 months compared to children aged 6–23 months, but also significant positive correlation in quality measures among these age groups at the survey level, with the important implication that surveys with more diligent, better trained and supervised measurers tend to produce higher quality of measurements irrespective of age group. The second implication of paramount importance is the need to improve the quality of measurements in the younger age group to bring it on par with the older age group. Therefore, it is important to ascertain that the key pre-survey training exercise for the measurers, standardization test [17] that includes 10 children measured twice by each survey team, must include an ample number of children from the younger age group (a minimum of 4, preferably 5 out of 10 total) so that the measurers get an ample opportunity to practice, and be evaluated, on recumbent length measurements and handling very young children who tend to be more restless and irritable and therefore more difficult to bring in correct measurement position compared to their older peers. The difference in quality of measurements in these age groups is not inherent or insurmountable, as demonstrated by a sizeable proportion (5–25% depending on indicator) of surveys in our dataset where the quality of measurements was slightly higher in younger than older age group.

## Supplementary information

**Additional file 1: Supplementary File.** Surveys and children by region.

## Data Availability

The data that support the findings of this study are available from Action Contre la Faim and the United Nations High Commissioner for Refugees but restrictions apply to the availability of these data, which were used under license for the current study, and so are not publicly available. Data are however available from the authors upon reasonable request and with permission of Action Contre la Faim and the United Nations High Commissioner for Refugees.

## Abbreviations

ACF: Action Contre la Faim
BMI: Body mass index
BMIZ: Body mass index-for-age z-score
DHS: Demographic and Health Survey
DOB: Date of birth
DP: Digit preference
HAZ: Height-for-age z-score
MUAC: Mid-upper arm circumference
MUACZ: Mid upper arm circumference -for-age z-score
SD: Standard deviation
UNHCR: United Nations High Commissioner for Refugees
UNICEF: United Nations Children’s Fund
WAZ: Weight-for-age z-score
WHO: World Health Organization
WHZ: Weight-for-height z-score
