# Supplementary material for: Comparison of anthropometric data quality in children aged 6-23 and 24-59 months: lessons from population-representative surveys from humanitarian settings
Source: BMC Nutr. 2020 Nov 13;6:60. doi: 10.1186/s40795-020-00385-0 (PMC7664017; doi:10.1186/s40795-020-00385-0)
Supplement: Supplementary file 1 — Additional file 1: Supplementary File. Surveys and children by region. [file 40795_2020_385_MOESM1_ESM.docx]

**Supplementary File. Surveys and children by region**

| **Regions** | **N Surveys** | **N Surveys with MUACZ** | **N Children**  **6 – 23 months** | **N Children**  **24 – 59 months** |
| --- | --- | --- | --- | --- |
| Latin America and the Caribbean | 13 | 13 | 1,868 | 3,281 |
| Eastern and Southern Africa | 335 | 325 | 56,950 | 101,826 |
| West and Central Africa | 217 | 217 | 47,337 | 85,464 |
| East Asia and Pacific | 122 | 122 | 2,194 | 4,020 |
| South Asia | 12 | 12 | 27,101 | 47,810 |
| Middle East and North Africa | 13 | 13 | 2,105 | 3,3633 |
| **TOTAL** | **712** | **702** | **137,555** | **246,034** |
